# Molecular characterization of duck enteritis virus UL41 protein

**DOI:** 10.1186/s12985-018-0928-4

**Published:** 2018-01-15

**Authors:** Tianqiong He, Mingshu Wang, Xuelian Cao, Anchun Cheng, Ying Wu, Qiao Yang, Mafeng Liu, Dekang Zhu, Renyong Jia, Shun Chen, Kunfeng Sun, Xinxin Zhao, Xiaoyue Chen

**Affiliations:** 1 Institute of Preventive Veterinary Medicine, Sichuan Agricultural University, Wenjiang, 611130 People’s Republic of China; 2 Avian Disease Research Center, College of Veterinary Medicine of Sichuan Agricultural University, Wenjiang, 611130 People’s Republic of China; 3 Key Laboratory of Animal Disease and Human Health of Sichuan Province, Sichuan Agricultural University, Wenjiang, 611130 People’s Republic of China

**Keywords:** DEV UL41, DEV UL47, Prokaryotic expression, γ2 gene, Purified virions, Mass spectrometry, Intracellular localization, NLS signals

## Abstract

**Background:**

Duck enteritis virus (DEV) belongs to the subfamily Alphaherpesvirinae, and information on the DEV UL41 gene is limited.

**Methods:**

The DEV UL41 gene was cloned into the pET32a(+) vector and expressed in a prokaryotic expression system. Antiserum was raised against a bacterially expressed UL41-His fusion protein for further experiments. Transcription was quantified and UL41 protein expression levels were determined in DEV-infected cells at different time points by RT-qPCR and western blotting, respectively. DEV virions were purified by sucrose gradient centrifugation and analyzed by mass spectrometry to identify protein content. We confirmed the DEV UL41 gene kinetic class using a pharmacological test. IFA was used to analyze the intracellular localization of pUL41.

**Results:**

The recombinant expression plasmid, pET-32a(+)-UL41, which highly expresses a 76.0 kDa fusion protein, was constructed and expressed in *E. coli* BL21 (DE3) after induction with 0.2 mM IPTG at 30 °C for 10 h, generating a specific mouse anti-UL41 protein polyclonal antibody. RT-qPCR and western blot analyses revealed that the UL41 transcript number peaked at 36 hpi, and peak protein expression occurred at 48 hpi. The pharmacological test showed that UL41 was a γ2 gene. Mass spectrometry analysis showed that pUL41 was a virion component. IFA results revealed that pUL41 was localized throughout DEV-infected cells but only localized to the cytoplasm of transfected cells. DEV pUL47 translocated pUL41 to the nuclei of DEF cells; this translocation was dependent on predicted pUL47 NLS signals (40–50 aa and 768–777 aa).

**Conclusions:**

DEV UL41 is a γ2 gene that encodes a virion structural protein, pUL41 localizes throughout DEV-infected cells but only localizes to the cytoplasm of transfected cells. pUL41 cannot autonomously localize to the nucleus, as this nuclear localization is dependent on predicted DEV pUL47 NLS signals (40–50 aa and 768–777 aa).

## Background

Duck plague (or duck viral enteritis) caused by the duck enteritis virus (DEV) is an acute, febrile, septic disease of waterfowl (ducks, geese, and swans of all ages and species) that leads to sizeable economic losses worldwide in the avian industry due to the associated high mortality rate and decreased duck egg production [1–5]. DEV belongs to the Alphaherpesvirinae subfamily, with a genome consisting of linear, double-stranded DNA comprising a unique long (UL) region, a unique short (US) region, a unique short internal repeat (IRS) region, and a unique short terminal repeat (TRS) region. The genomic arrangement pattern is UL-IRS-US-TRS [6].

The UL41 gene is highly conserved in alphaherpesviruses [7, 8], and UL41-encoded proteins have been described in other Alphaherpesvirinae subfamily members, including bovine herpesvirus 1 (BHV-1) [9, 10], monkey B virus (macacine herpesvirus 1; BV) [11], equine herpesvirus-1 (EHV-1) [12], pseudorabies virus (PRV) [7], and varicella-zoster virus (VZV) [13, 14]. The HSV-1 virion host shutoff (VHS) protein, a late (γ2) tegument protein, is encoded by the UL41 gene and is packaged into the virion; essential for viral infection [15–19], VHS is released into the host cell cytoplasm, selectively degrading cellular mRNAs via its RNase activity and contributing to the shutoff of host cell protein synthesis early in infection [20, 21]. In addition, VHS-RNase activity is tightly modulated by several HSV proteins. Early in the infection, VHS-RNase first binds tegument proteins VP13/14 and ICP27, encoded by UL47 and UL54 genes, respectively, and selective degradation of mRNAs by the VHS-RNase is regulated by the VP13/14. Then, late in the infection, VHS-RNase activity is neutralized by VP16 and VP22, encoded by UL48 and UL49 genes, respectively [21].VHS functions in BHV-1 [9], BV [10] and EHV-1 [12] may serve the same purpose in the mechanisms of these viruses. The PRV VHS protein also exhibits RNase activity, but it is not identical to that of HSV-1 VHS [7, 22]. VZV open reading frame 17 (ORF17) is a late nonstructural protein with a delayed cellular RNA shutoff and a homologue of the VHS protein but has no major function in VZV-mediated delayed host shutoff [13, 14].

The characteristics of some DEV genes have been reported [23–35]; however, information regarding the DEV UL41 gene is limited. Bioinformatic analyses have revealed that the full-length sequence of the DEV UL41 ORF is 1494 bp and encodes a 497 amino acid protein with a molecular mass of 56 kDa and shares 31% homology with the HSV-1 VHS protein. In this study, we characterized the DEV UL41-encoded protein (pUL41) using western blot, real-time quantitative reverse-transcription PCR (RT-qPCR), indirect immunofluorescence assays (IFAs), a pharmaceutical test and mass spectrometry. Our results will provide guidance for further study of DEV UL41.

## Methods

### Viruses, cells and vectors

The DEV CHV strain (Gene bank: JQ647509.1) was isolated and preserved in our laboratory [22].

Duck embryo fibroblasts (DEF) were propagated in minimal essential medium (MEM; Gibco, Meridian Road Rockford, USA) supplemented with 10% (*v*/v) fetal bovine serum (FBS; Gibco, Meridian Road Rockford, USA) at 37 °C with 5% CO_2_. For viral infections, maintenance medium supplemented with 2% FBS was added. Commonly used reagents were prepared in our laboratory.

The recombinant β-actin plasmid [32] and rabbit anti-UL47 protein polyclonal antibody were prepared in our laboratory.

### Construction of the recombinant expression vector

All primers were designed by Primer Premier 5 software (Table 1). The wild-type DEV UL41 (GenBank: AFC61841.1) coding region was constructed in the pET-32a(+) vector (Novagen, Podenzano, Italy) using specific primers (P_1_ and P_2_) to create pET-32a(+)-UL41, and in the eukaryotic plasmid pEGFP-C2 using specific primers (P_3_ and P_4_) to create pEGFP-C2-UL41. The wild type DEV UL47 (GenBank: AFC61835.1) coding region was sub-cloned into pcDNA3.1(+) using specific primers (P_5_ and P_6_), creating pcDNA3.1(+)-UL47. Next, we constructed the eukaryotic plasmids pcDNA3.1(+)-UL47_Δ40–50_ and pcDNA3.1(+)-UL47_Δ768–777,_ which carry deletions from 40 to 50 and 768 to 777 amino acids (aa) of the DEV UL47-encoded protein (pUL47), respectively.

**Table 1:** Sequences and primer pair characteristics

Primer	Primer sequence (5′ → 3′)	Gene	Product size (bp)
P_1_	G/AATTCATGGGGCTGTATGGTTGTATAAGC	DEV UL41	1526
P_2_	C/TCGAGTCTTACAACAGTTAATCTTAGTCCCAAT		
P_3_	G/AATTCGCCACCATGGGGCTGTATGGTTGTATAAGC	DEV UL41	1532
P_4_	G/GTACCTCTTACAACAGTTAATCTTAGTCCCAAT		
P_5_	CGCG/GATCCGCCACCATGGATAAATCACGAAGACAGCG	DEV UL47	2391
P_6_	CCGC/TCGAGTTAATGTAACTCTCTCCGCCCAG		
P_7_	TGATTTACACCGCTACCCTA	DEV UL41(RT-PCR)	107
P_8_	TCTCACTTCTTTCAGCCATT		
P_9_	CCGGGCATCGCTGACA	Duck β-actin(RT-PCR)	177
P_10_	GGATTCATCATACTCCTGCTTGCT		
P_11_	GAACAACCGCCGAACAC	DEV UL54	127
P_12_	TCAAACATCCGCCTCAA		
P_13_	GCCACCAACCCTACCAAG	DEV UL13	131
P_14_	GTCGTCAGCCCATCACCA		
P_15_	AGACGGTTCCGAAAGTACAG	DEV Us2	111
P_16_	TCGGCAGCACCAATAATCC		

### Prokaryotic expression of UL41-his fusion proteins in *E. coli*

pET-32a(+)-UL41 was transformed into *E. coli* BL21 (DE3)-competent cells. Bacterial cultures were kept at 37 °C in Luria-Bertani (LB) medium containing 50μg/ml ampicillin. Protein expression was induced by adding IPTG (St Louis, MO, USA). Different induction concentration effects (0.1, 0.2, 0.4, 0.6, 0.8, 1.0, and 1.2 mM), induction times (2, 4, 6, 8 and 10 h) and induction temperatures (25, 30 and 37 °C) were examined to optimize conditions to obtain the highest level of UL41 protein expression [33]. After IPTG induction, bacteria were collected at different time points by centrifugation at 4 °C and disrupted by ultrasonication. The vector control culture was analyzed in parallel. All expression levels were analyzed by sodium dodecyl sulfate-polyacrylamide gel electrophoresis (SDS-PAGE) with 12% resolving gels and 5% stacking gels.

### Purification of the fusion protein and preparation of the polyclonal antibody

The UL41 protein was purified by gel and electric elution, and the purified protein was used to generate a polyclonal antibody in mice. Approximately 1.1 mg of purified protein mixed with an equal volume of QuickAntibody-Mouse3W adjuvant (Biodragon, Beijing, China) was used to immunize eleven mice by intramuscular injection. Then, the mice were exsanguinated by eyeball removal 1 week after the last immunization, and antisera were harvested by centrifugation (9600×*g*, 20 min, 4 °C).

### Western blotting

As previously described [29], DEV-infected DEF cells in 6-well dishes were collected at 7, 24, 36, 48, 60, 72 h post-infection (hpi), removing the supernatant. Proteins that were resolved by 12% SDS-PAGE were further transferred to polyvinylidene fluoride (PVDF) membranes. Membranes were blocked for 2 h in 5% skim milk, then incubated with primary antibody and probed with HRP-conjugated secondary antibody (Bio-rad Lab, CA, USA) for 1 h at 37 °C. All antibodies were diluted in 1% skim milk. After several washes with PBST to remove unbound antibodies, the signal was detected using Western BLoT Chemiluminescence HRP Substrate (Takara, Dalian, China) according to the manufacturer’s instructions.

### Indirect immunofluorescence assay (IFA)

As previously described [35], DEV-infected DEF cells in 6-well dishes were collected on coverslips at 0, 7, 12, 24, 36, 48, 60, 72 hpi, fixed with 4% paraformaldehyde in PBS for 30 min, permeabilized with 0.25% Triton X-100 in PBS for 30 min at 4 °C and blocked for 1 h with 5% BSA PBS at 37 °C. Additionally, cells were incubated for 1 h with primary antibodies, then secondary antibodies (Thermo Fisher Scientific, Meridian Road Rockford, USA). All antibodies were diluted in 1% BSA PBS. Finally, the cell nuclei were visualized with DAPI (Roche, Mannheim, Germany)for 15 min at room temperature. Coverslips were sealed on glass slides with glycerin buffer, and the cells were examined using a confocal microscope (Nikon A1, Japan).

### RT-qPCR

Total RNA from the DEV-infected DEF cells at different time points (0, 2, 6, 12, 24, 36, 42, 45, 48, and 54 hpi) was extracted using TRIzol reagent l (Invitrogen, CA, USA) according to the manufacturer’s recommendations. Extracted RNA integrity and purity were evaluated by 1% agarose gel electrophoresis and by optical density measurements (OD260/OD280 ratio) using a NanoDrop spectrophotometer, respectively. Subsequently, the RNA was reverse transcribed into cDNA using the PrimeScript®RT reagent kit with the gDNAeraser (Takara, Beijing, China). The UL41 (P_7_ and P_8_) and β-actin (P_9_ and P_10_) [35] genes were detected using the previously described primers (Table 1). The RT-qPCR conditions were set as follows: initial denaturation at 95 °C for 1 min, followed by 45 cycles of denaturation at 95 °C for 5 s, annealing at 59 °C for 20 s, and extension at 72 °C for 25 s. All reactions were performed in triplicate and in at least two independent experiments. The cycle number at threshold (Ct value) was determined to analyze UL41 and β-actin gene transcription, and the results were calculated using the 2^-ΔΔCT^ method [25].

### Pharmaceutical identification virion gene type

DEV-infected DEF cells cultured in 6-well dishes were incubated with 300 μg/ml acyclovir (Glaxo SmithKline) or 50 μg/ml cycloheximide (Meilunbio, Dalian, China) for 2 h. Total RNA was extracted at 24 hpi with TRIzol and subsequently reverse transcribed into cDNA. PCR was conducted using primers for UL41 (P_1_ and P_2_) and β-actin (P_9_ and P_10_) and confirmed by 1% agarose gel. The immediate-early (IE) gene UL54 (P_11_ and P_12_) [35], early (E) gene UL13 (P_13_ and P_14_) [36], and late (L) gene Us2 (P_15_ and P_16_) [37] were detected as experimental controls.

### Virion purification

DEV-infected DEF cells were collected, and cellular debris was removed by low-speed centrifugation (2000×*g*, 30 min, 4 °C). Extracellular DEV virions were harvested by ultracentrifugation (40,000×*g*, 2 h, 4 °C) in a Beckman Ti70 rotor through a 30% (wt/vol) sucrose cushion. Viral bands were collected by isopycnic gradient ultracentrifugation in a continuous 30 to 60% (wt/vol) potassium tartrate gradient in TBS (40,000×*g*, 2 h, 4 °C) in a Beckman SW60 rotor, diluted ten-fold in TBS and then pelleted by ultracentrifugation (20,000×*g*, 60 min, 4 °C). The pellet was finally resuspended in TBS and stored at − 80 °C.

### Mass spectrometry

Purified virion samples were analyzed by 12% SDS-PAGE. The gel was stained with Coomassie brilliant blue (Sigma) and sent to Sangon Biotech Company (Shanghai, China) for liquid chromatography-tandem mass spectrometry (LC-MS/MS) analysis [38]. All data were also searched against the NCBI *Bos taurus* database. Only sequences identified with a Mascot score greater than 30 were considered. Proteins identified by at least one unique peptide were accepted. And the exponentially modified protein abundance index (emPAI) was used to estimate protein relative abundance for the complete virion extracts [39–41].

## Results

### Construction of the recombinant expression vector

PCR reactions were performed using specific primers and the DEV CHV strain sequence. The full length of UL41 was cloned into the pMD19-T vector, then subcloned into the pET-32a(+) expression vector and eukaryotic plasmid pEGFP-C2, referred to as pET-32a(+)-UL41 and pEGFP-C2-UL41, respectively. Briefly, the two mutant UL47s with 40–50 aa “RRSGKRRTLDR” (Δ40–50) and 768–777 aa “KALKRRLTGG” (Δ768–777) mutations were obtained by fusion PCR. The wild-type UL47 and two mutants were sub-cloned in the pcDNA3.1(+) vector, creating the resulting plasmids, named pcDNA3.1(+)-UL47, pcDNA3.1(+)-UL47_Δ40–50_ and pcDNA3.1(+)-UL47_Δ768–777_, respectively.

### Preparation of the mouse anti-UL41 protein polyclonal antibody

**Fig. 1:**
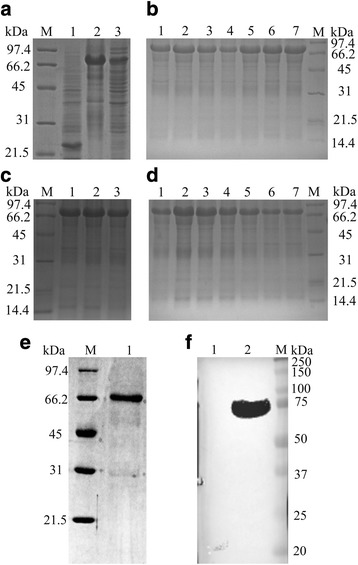
Purification of the fusion protein and preparation of the polyclonal antibody. All bacteria samples were collected by centrifugation at 4 °C and disrupted by ultrasonication, and then analyzed by SDS-PAGE with 12% resolving gels and 5% stacking gels. **a** Lane 1, pET-32a(+) vector; Lane 2, recombinant bacterial sediment; Lane 3, recombinant bacterial supernatant; M. Bio-Rad low-molecular-weight protein marker. Protein expression was induced at 37 °C for 6 h with 0.4 mM IPTG until OD_600_ reached 0.4–0.6. Significantly higher UL41-His recombinant protein levels were observed in the recombinant bacterial sediment. **b** IPTG concentration optimization. Lanes 1–7, IPTG concentrations of 1.2, 1.0, 0.8, 0.6, 0.4, 0.2, and 0.1 mM, respectively. M. Bio-Rad low-molecular-weight protein marker. Significantly higher UL41-His recombinant protein levels were observed at 0.2 mM. **c** Induction temperature optimization. Lane 1, induction at 25 °C; Lane 2, induction at 30 °C; Lane 3, induction at 37 °C; M. Bio-Rad low-molecular-weight protein marker. Significantly higher UL41-His recombinant protein levels were observed at 30 °C. **d** Induction time optimization. Lanes 1–7, induction for 12.5, 10, 8, 6, 4, 2, and 1 h, respectively; M. Bio-Rad low-molecular-weight protein marker. Significantly higher UL41-His recombinant protein levels were observed at 10 h. **e** Recombinant protein purification. Lane 1, purified protein (approximately 76 kDa); M. Bio-Rad low-molecular-weight protein marker. **f** Expressed protein identification by western blotting using rabbit anti-DEV antibody (1:800) and goat anti-rabbit IgG HRP-conjugated antibody (1:5000). Lane 1, pET-32a(+) vector; Lane 2, recombinant protein (approximately 76 kDa); M. Precision Plus Protein™ Dual Color Standards

The recombinant pET-32a(+)-UL41 plasmid expressing DEV UL41 fused with a 6-histidine tag was transformed into *E. coli* BL21 (DE3). The SDS-PAGE results detected an obvious band (approximately 76 kDa) exclusively in the insoluble fraction and undetected in the vector control culture (Fig. 1a). The optimal protein expression level was induced using 0.2 mM IPTG (Fig. 1b) at 30 °C (Fig. 1c) for 10 h (Fig. 1d).

**Fig. 2:**
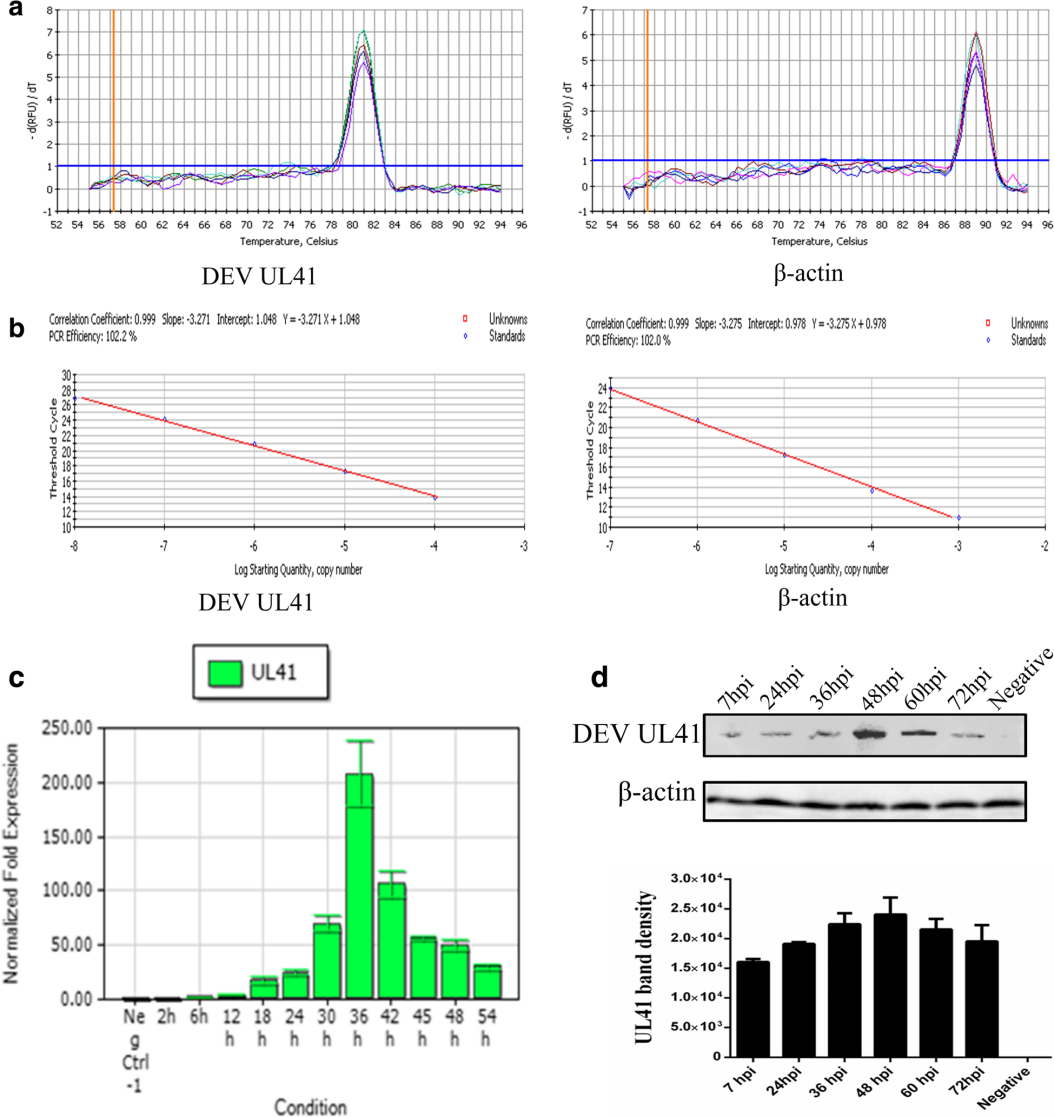
The transcription and expression kinetics of the DEV UL41 gene. **a** Melting curves of DEV UL41 (81 °C) and β-actin (89 °C). **b** Standard curves of DEV UL41 (Y = − 3.271X + l.048) and β-actin (Y = − 3.275X + 0.978), and UL41 gene (102.2%) and β-actin (102.0%) amplification efficiencies were approximately identical, with correlation coefficients of 0.999. **c** UL41 mRNA levels at 0, 2, 6, 12, 24, 36, 42, 45, 48, and 54 hpi were assessed by RT-qPCR and normalized toβ-actin. The UL41 transcript was detected at 6 hpi, increased gradually (6–30 hpi), reached its peak at 36 hpi, and then decreased steadily thereafter (42–54 hpi). **d** Representative western blotting results for UL41 at 7, 24, 36, 48, 60, and 72 hpi (up) (approximately 56 kDa) and quantification in the six groups (down). The negative group comprised mock-treated DEF cells detected with mouse anti-UL41 antibody serum (1:800) and goat anti-mouse IgG HRP-conjugated antibody (1:5000). The band density increased gradually (7-36hpi), peak expression occurred at 48 hpi, and expression decreased steadily thereafter (60–72 hpi). β-actin antibody was used to assess β-actin as the loading control

Next, the fusion protein was purified by gel and electric elution (Fig. 1e) and detected using rabbit anti-DEV antibody by western blotting (Fig. 1f). The purified protein was used to generate the mouse anti-UL41 protein polyclonal antibody, which reacted strongly with the UL41 protein in the DEV-infected cells, showing a band with a mobility of approximately 56 kDa (Fig. 2d).

### Transcription kinetics of the DEV UL41 gene

RT-qPCR was used to identify the transcription kinetics of the DEV UL41 gene with SYBR Green I. The melting curves showed that the specificities of the primers were excellent (Fig. 2a). Standard curves for the UL41 and β-actin genes were established to evaluate the efficiency of the assays (Fig. 2b). Subsequently, total RNA from the DEV-infected cells was collected at different times, reverse transcribed to cDNA, and subjected to RT-qPCR; the results were determined using the 2^-ΔΔCT^ method. As shown in Fig. 2c, the UL41 gene transcript was detected at 6 hpi, and the transcript level increased gradually, reached its peak at 36 hpi, and then steadily decreased.

### Expression levels of the DEV UL41 gene

Western blot analysis was performed on the cell lysates collected at different time points post-infection, and the membranes were probed with the mouse anti-UL41 protein polyclonal antibody (Fig. 2d). Band density increased gradually, and peak expression occurred at 48 hpi, decreasing steadily thereafter. The β-actin antibody was used to assess β-actin as the loading control due to its low turnover rate, and the β-actin levels remained relatively constant. Band density was analyzed using ImageJ software.

### DEV UL41 is a late gene

**Fig. 3:**
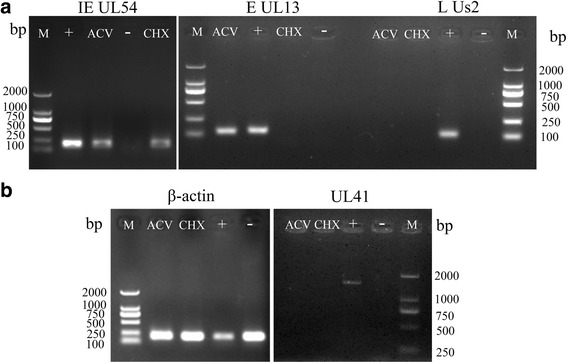
Pharmaceutical identification virion gene type. **a** M, DL2000 Marker; ACV group: DEV-infected cells treated with 300 μg/ml acyclovir (ACV); CHX group: DEV-infected cells treated with 50 μg/mL cycloheximide (CHX). (+) group: DEV-infected cells without drugs; (−) group: untreated DEF cells. The DEV IE gene UL54 (127 bp), E gene UL13 (131 bp), and L gene Us2 (111 bp) are shown, respectively. **b** M, DL2000 Marker; housekeeping gene β-actin (177 bp) and DEV UL41 (1526 bp) are shown, respectively

We treated the infected cells with acyclovir (ACV) or cycloheximide (CHX). The correct bands of the IE gene UL54, E gene UL13, and L gene Us2 are shown (Fig. 3a). The β-actin band (Fig. 3b) was detected in the control group (infected, untreated cells), the negative group (untreated DEF cells) and the ACV and CHX groups. The UL41 band was detected in the control group but not in the negative, ACV or CHX groups (Fig. 3b). The results showed that DEV UL41 is an L gene.

### UL41 protein is a virion component

We identified the extracellular virion protein content by mass spectrometry. The results showed that the UL41 protein was present in mature extracellular virions. Two unique DEV UL41 peptides were detected, while three unique peptides matched DEV gC(*P < 0.05*). The relative abundance of UL41 was low based on the exponentially modified protein abundance index (emPAI) (Table 2).

**Table 2:** Viral content of DEV extracellular virions (partial)

Protein	Information	Score	Mass	Matches	Sequences	emPAI	NCBI Accession
UL44	glycoprotein C	97	47,836	6(3)	6(3)	0.22	AJG04885
UL41	virion protein	46	57,546	6(2)	6(2)	0.12	AJG04888

### DEV UL41 protein mainly localizes to the cytoplasm in newly infected cells

**Fig. 4:**
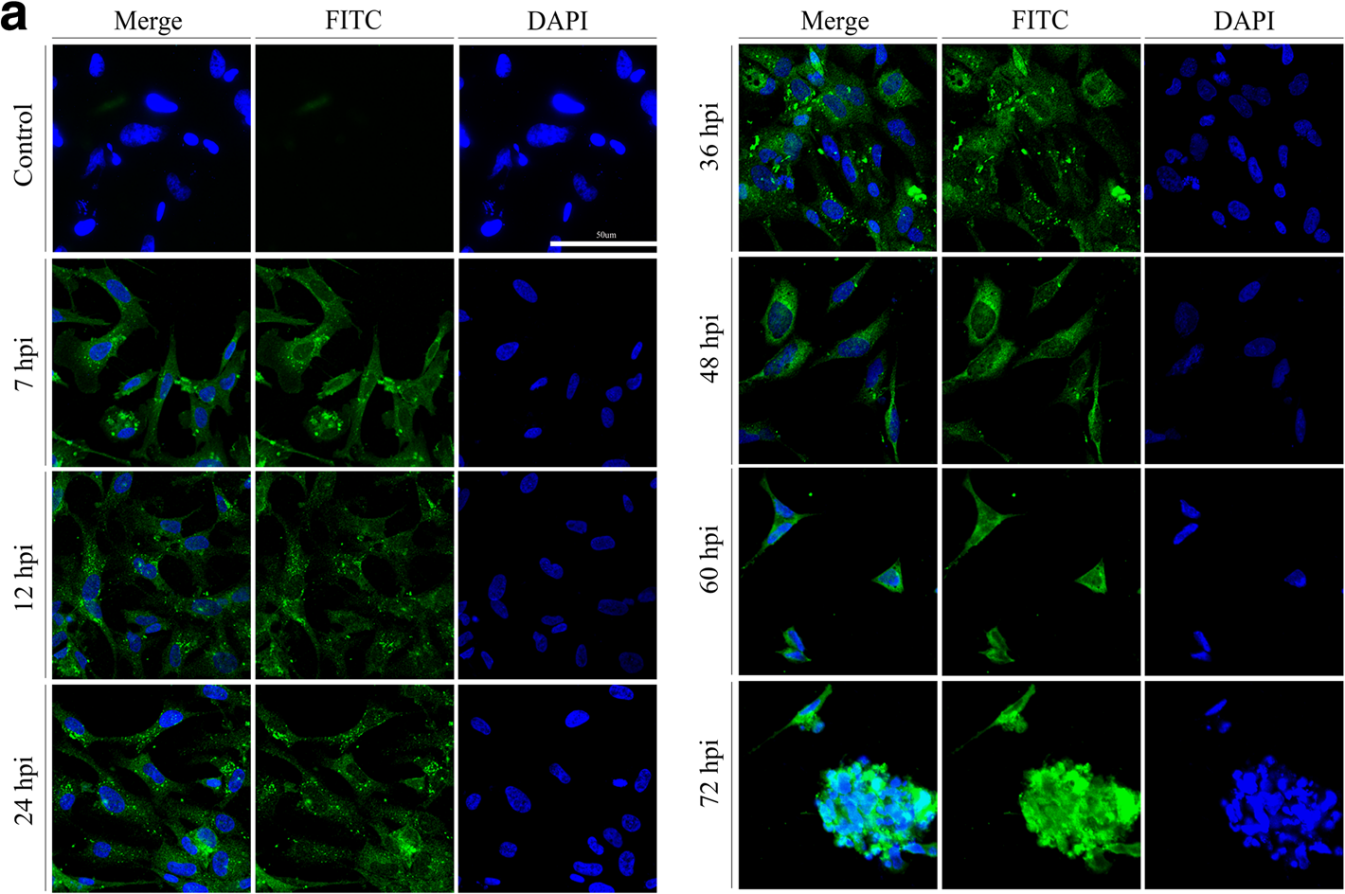
pUL41 intracellular localization in DEV-infected cells at different times. **a** Representative confocal immunofluorescence microscopic images of the UL41 localization (green in all images) at 7, 12, 24, 36, 48, 60, and 72 hpi. Nuclei are indicated in blue (DAPI). The control group comprised untreated DEF cells (magnification: 200×; scale bar: 50 μm) detected with mouse anti-UL41 antibody serum (1:800) and goat anti-mouse IgG (H + L) cross-adsorbed secondary antibody, Alexa Fluor 488 (1:1000). Significantly higher UL41 levels in the cytoplasm were evident in the DEF cells infected with wild-type DEV

IFA was used to confirm the intracellular localization of the DEV UL41 protein (pUL41). Generally, we found that UL41 protein-specific fluorescence (green) was distributed throughout the cells and was mainly evident in the cytoplasm. Additionally, UL41-specific fluorescence was evident in the cells between 7 and 36 hpi, subsequently becoming scattered throughout the cell between 48 and 72 hpi, then gradually becoming stronger over time. No fluorescence was observed in the mock-infected cells (Fig. 4a).

### DEV UL47 protein may translocate pUL41 into the nucleus in the absence of other viral proteins

**Fig. 5:**
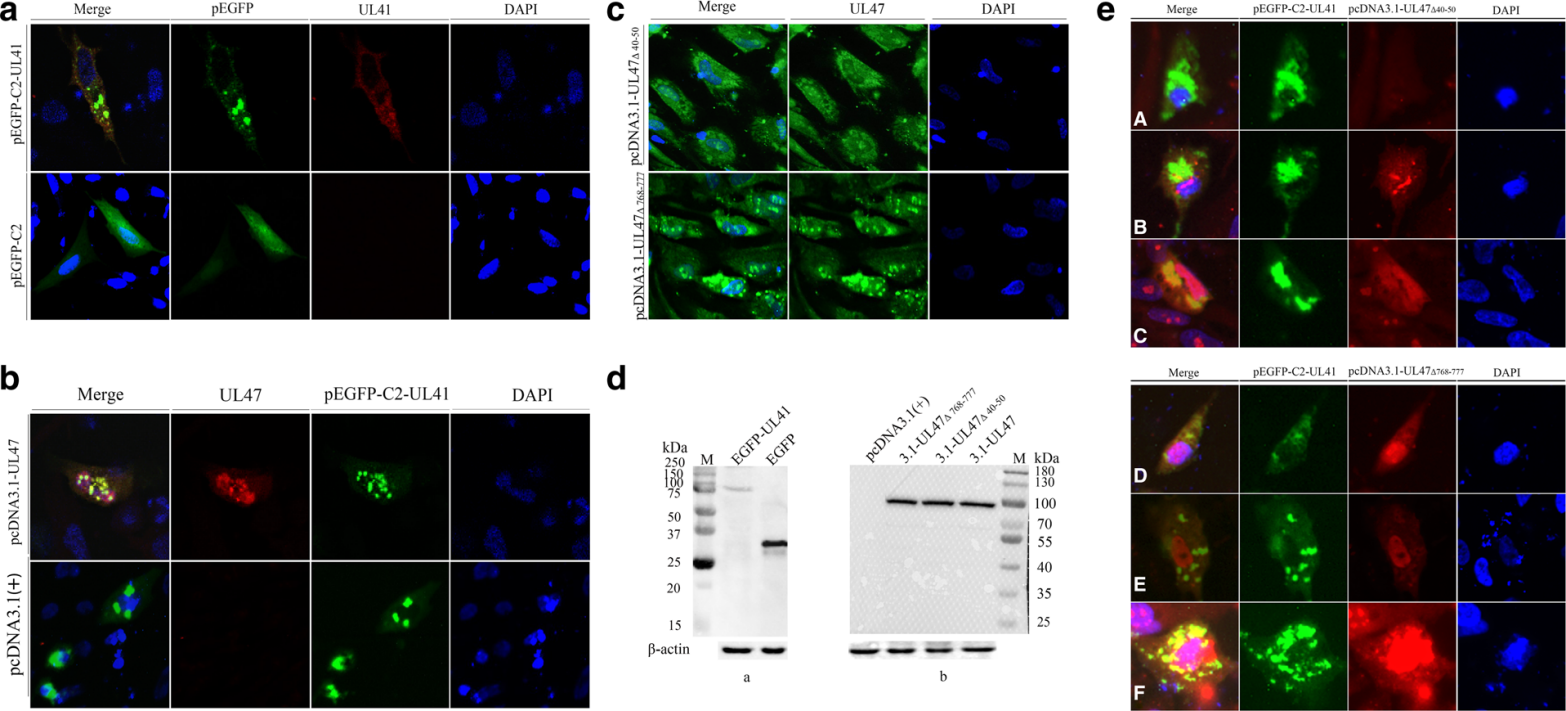
DEV-UL47 protein may translocate pUL41 into the nucleus in the absence of other viral proteins. All transfected samples were collected after 24 h post transfection. **a** DEF cells were transfected with pEGFP-C2-UL41 and pEGFP-C2, respectively. The EGFP-UL41 fusion protein was localized to the cytoplasm (green), further detected with mouse anti-UL41 protein polyclonal antibody (1:800) and goat anti-mouse IgG (H + L) cross-adsorbed secondary antibody, Alexa Fluor 568 (1:1000) (red). The pEGFP-C2 plasmid control was distributed throughout the cells (green). **b** DEF cells were co-transfected with pEGFP-C2-UL41 and pcDNA3.1-UL47. Much of the EGFP-UL41 fusion protein was localized to the nucleus (green), and pUL47 was mainly localized to the nucleus (red), detected with the rabbit anti-UL47 protein polyclonal antibody (1:1000) and goat anti-rabbit IgG (H + L) cross-adsorbed secondary antibody, Alexa Fluor 568 (1:1000). DEF cells were co-transfected with pEGFP-C2-UL41 and pcDNA3.1(+) as the control, and the EGFP-UL41 fusion protein was only distributed in the cytoplasm (green). **c** DEF cells were transfected with pcDNA3.1-UL47_Δ40–50_ and pcDNA3.1-UL47_Δ768–777_, respectively. A portion of the ΔpUL47 was localized to the cytoplasm (green), detected with rabbit anti-UL47 protein polyclonal antibody (1:1000) and goat anti-rabbit IgG (H + L) cross-adsorbed secondary antibody, Alexa Fluor 488 (1:1000). **d** Representative western blotting results for DEF cells transfected with pEGFP-C2-UL41 (approximately 83 kDa) and pEGFP-C2 (approximately 27 kDa), detected with rabbit anti-EGFP antibody (1:2000) (Beyotime, Shanghai, China) and goat anti-rabbit IgG HRP-conjugated antibody (1:5000). Western blotting results for DEF cells transfected with pcDNA3.1(+), pcDNA3.1-UL47_Δ768–777_ (approximately 87 kDa), pcDNA3.1-UL47_Δ40–50_ (approximately 87 kDa) and pcDNA3.1-UL47 (approximately 89 kDa), respectively, detected with rabbit anti-UL47 antibody (1:1000) and goat anti-rabbit IgG HRP-conjugated antibody (1:5000). The β-actin antibody assessed β-actin as the loading control. M. Precision Plus Protein™ Dual Color Standards. **e** DEF cells were co-transfected with pEGFP-C2-UL41 and pcDNA3.1-UL47_Δ40–50_, pEGFP-C2-UL41 and pcDNA3.1-UL47_Δ768–777_, respectively. Much of the EGFP-UL41 fusion protein was localized to the cytoplasm (green). A portion of ΔpUL47 was localized to the cytoplasm (red), detected with rabbit anti-UL47 protein polyclonal antibody (1:1000) and goat anti-rabbit IgG (H + L) cross-adsorbed secondary antibody, Alexa Fluor 568 (1:1000)

The eukaryotic plasmid, pEGFP-C2-UL41, expressed the 83 kDa fusion protein, EGFP-UL41 (Fig. 5d), which was only localized to the cytoplasm (green) and further detected by mouse anti-UL41 protein polyclonal antibody (red). The pEGFP-C2 plasmid control was distributed throughout the cells (Fig. 5a).

According to our previous study, pUL41 was distributed throughout infected cells, so we hypothesized that another DEV protein may affect this localization. Another HSV tegument protein, pUL47, has been reported to interact with VHS, enabling nuclear localization of VHS [21, 42]. To determine whether the DEV pUL47 would translocate pUL41 into the nucleus, we constructed the eukaryotic plasmid pcDNA3.1-UL47, which expresses the 89 kDa pUL47 (Fig. 5d). DEF cells were co-transfected with the eukaryotic plasmids pcDNA3.1-UL47 and pEGFP-C2-UL41, and pUL47 was primarily localized to the nuclei of DEF cells (red), as detected by the rabbit anti-UL47 protein polyclonal antibody (1:1000). The EGFP-UL41 fusion protein was also primarily identified in the nuclei (green) (Fig. 5b). Thus, we concluded that pUL47 may translocate pUL41 to the nucleus.

Per the prediction software at https://www.predictprotein.org/, DEV pUL47 has two evident nucleus localization signals (NLSs), from 40 to 50 aa and from 768 to 777 aa. To identify whether the two pUL47 sequences translocated pUL41 into the nucleus, we constructed the plasmids, pcDNA3.1-UL47_Δ40–50_ and pcDNA3.1-UL47_Δ768–777_, which deleted the 40–50 aa and 768–777 aa of pUL47, respectively (Fig. 5d). The IFA showed that much of the ΔpUL47 was localized to the cytoplasm (green) (Fig. 5c); thus, we concluded that both the 40–50 aa and 768–777 aa affected pUL47 nucleus localization. Next, we co-transfected the pcDNA3.1-UL47_Δ40–50_ and pEGFP-C2-UL41, and pcDNA3.1-UL47_Δ768–777_ and pEGFP-C2-UL41, respectively. In two co-transfected cells, the EGFP-UL41 fusion protein was primarily localized to the cytoplasm (green) (Fig. 5e).

## Discussion

Until now, information regarding the DEV UL41 gene has been limited. Jinxiong Liu and colleagues [43] constructed a recombinant virus, rDEV-ul41HA, to serve as a bivalent live-attenuated vaccine against both DEV and H5N1 infections in ducks, using the UL41 gene of the DEV genome as the insertion site. UL41 in other herpesvirus genomes was identified as a region nonessential for viral replication [44]; however, the results indicated that UL41 was not an ideal site for foreign gene insertion. We further explored the function of the DEV pUL41 as the first step in studying the properties and characteristics of the DEV UL41 protein, and the salient features of the results presented here were as follows.

The herpesvirus cycle is characterized by two distinct phases: latency and the productive or lytic cycle. During the productive cycle, herpesviruses exhibit strictly regulated temporal cascades of gene expression that are divided into three stages: IE, E, and L. IE genes are expressed first and encode nuclear regulatory proteins that act at the transcriptional and post-transcriptional levels to stimulate viral E and L gene expression. Second, E genes participate in viral DNA replicative machinery. Viral DNA replication then augments expression of L genes that encode most of the structural components of the viral particles. L genes are subdivided into two categories as leaky late (γ1) or strict late (γ2) based on their apparent DNA replication requirements. The expression of γ1 genes is delayed compared to that of early genes, whereas γ2 gene strictly requires the onset or completion of lytic DNA amplification [45, 46]. The HSV-1 VHS protein is a γ2 tegument protein that contributes to the degradation of cellular messenger RNAs (mRNAs) and an overall decrease in host protein synthesis [20, 21]. Additionally, VZV ORF17 is also expressed as an L protein that shuts off cellular RNA during the viral replication cycle [14]. The UL41 gene of the infectious laryngotracheitis virus (ILTV) is classified as an E/L gene because it has both E and L properties [47].

To identify the DEV UL41 gene type, we studied its transcription and expression kinetics at different time points post-infection using RT-qPCR and western blot analysis, respectively. Based on previous studies [32, 37, 48, 49], we suggested that accumulation of the DEV pUL41 may occur during the late stage of infection. IE genes are expressed in the presence of CHX, a protein synthesis inhibitor, while transcription of E and L genes is suppressed in the presence of CHX [50]. To investigate this further, we treated the infected cells with a DNA polymerase inhibitor, ACV, and a protein synthesis inhibitor, CHX, and observed that the UL41 gene was inhibited in both the ACV and CHX groups. Thus, the DEV UL41 gene was a γ2 gene and strictly dependent on the onset or completion of lytic DNA amplification. Mass spectrometry is an efficient means to identify protein composition of complex samples and has been performed in the analysis of various herpesviruses [39, 40, 51–53]. Based on the emPAI,DEV pUL41 is a low-abundance virion component, which means that DEV pUL41 may share identical characteristics with the HSV-1 VHS protein, which is essential for viral infection.

Intracellular viral protein localization is associated with viral protein function, but intracellular DEV pUL41 localization is undocumented. We analyzed DEV pUL41 intracellular localization by IFA using the mouse anti-UL41 protein polyclonal antibody. The results showed that DEV UL41 protein-specific fluorescence was distributed throughout the cells and that the signal was mainly present in the cytoplasm. According to previous studies [19, 45], the HSV-1 VHS protein is delivered to the cytoplasm of newly infected cells to degrade cellular mRNAs. This degradation process contributes to shutting off host protein synthesis, redirecting the cellular machinery from host to viral gene expression, and disrupting pre-existing polyribosomes. These processes comprise the major early mechanism through which the virus blocks the host’s response to infection. Studies by other investigators [21] have shown that the wild-type HSV-1 VHS protein localizes initially to the nucleus and then is translocated to the cytoplasm. The protein, which can shuttle between the nucleus and the cytoplasm, contains a putative nuclear export signal (NES), but no obvious NLS has been identified. Another late tegument HSV-1 protein, pUL47 (or VP13/14), which binds RNA and shuttles between the cytoplasm and the nucleus [54], can regulate subcellular localization of some viral and cellular proteins that interact with it, may be involved in translocating VHS to the nucleus [55–57]. VHS-RNase and VP13/14 must assemble with newly synthesized and processed mRNA in the nucleus prior to VHS-RNase/VP13/14 translocation to the cytoplasm, where VHS-RNase then degrades the mRNA. Early in the infection, VHS function is regulated by VP13/14, which attenuates degradation of all kinetic classes of viral mRNAs and stable host mRNAs in the infected cells with no apparent effect on the stability of AU-rich mRNAs [42]. Sequence prediction reveals that DEV UL41 contains NES motifs but no NLS motifs. Based on these reports, we also found that DEV pUL41 could not localize autonomously to the nucleus and that DEV pUL47 translocated pUL41 into the nucleus in the absence of other viral proteins; these processes were dependent on predicted pUL47 NLS signals.

From these data, we concluded that DEV UL41 is a γ2 gene that encodes a structural protein similar to HSV-1 VHS. DEV pUL41 may be essential for viral infection, functioning in the cytoplasm of infected cells and interacting with pUL47 to affect viral and host genes, but additional studies are required to confirm this.

## Conclusions

DEV UL41 is a γ2 gene that encodes a structural protein. UL41-encoded protein localizes throughout the DEV-infected cells, but pUL41 cannot localize autonomously to the nucleus, as this nuclear localization is dependent on the predicted NLS signals of DEV pUL47 (40–50 aa and 768–777 aa).

## Abbreviations

ACV: acyclovir
BHV-1: bovine herpesvirus 1
BV: Monkey B virus (Macacine herpesvirus 1)
CHX: cycloheximide
DAPI: 4′,6-diamidino-2-phenylindole
DEV: duck enteritis virus
EHV-1: equine herpesvirus-1
HSV: herpes simplex virus
IFAs: indirect immunofluorescence assays
ILTV: infectious laryngotracheitis virus
NES: nuclear export signal
NLS: nuclear localization signal
PRV: pseudorabies virus
PVDF: polyvinylidene fluoride
RT-PCR: real-time quantitative reverse-transcription PCR
SDS-PAGE: sodium dodecyl sulfate polyacrylamide gel electrophoresis
VZV: varicella-zoster virus
